# Therapeutic Opportunities in Colorectal Cancer: Focus on Melatonin Antioncogenic Action

**DOI:** 10.1155/2019/9740568

**Published:** 2019-09-17

**Authors:** Hucong Wu, Jiaqi Liu, Yi Yin, Dong Zhang, Pengpeng Xia, Guoqiang Zhu

**Affiliations:** ^1^College of Veterinary Medicine, Yangzhou University, Yangzhou 225009, China; ^2^Jiangsu Co-Innovation Center for Prevention and Control of Important Animal Infectious Diseases and Zoonoses, Yangzhou 225009, China

## Abstract

Colorectal cancer (CRC) influences individual health worldwide with high morbidity and mortality. Melatonin, which shows multiple physiological functions (e.g., circadian rhythm, immune modulation, and antioncogenic action), can be present in almost all organisms and found in various tissues including gastrointestinal tract. Notably, melatonin disruption is closely associated with the elevation of CRC incidence, indicating that melatonin is effective in suppressing CRC development and progression. Mechanistically, melatonin favors in activating apoptosis and colon cancer immunity, while reducing proliferation, autophagy, metastasis, and angiogenesis, thereby exerting its anticarcinogenic effects. This review highlights that melatonin can be an adjuvant therapy and be beneficial in treating patients suffering from CRC.

## 1. Introduction

Colorectal cancer (CRC) is the third most commonly diagnosed cancer and a major cause of cancer-related mortality around the world [1–3]. Multiple factors are associated with the occurrence and the development of CRC, including genetic makeup, population aging/gender, dietary behaviors, poor physical activity, and smoking [4–6]. According to the clinical situations of the patients with CRC, the status of CRC treatments (e.g., surgical therapy, radiotherapy, chemotherapy, targeted therapy, and immunotherapy) develops rapidly [7]. Even though different and novel therapies are available, in almost >25% of patients with metastatic cancer systemic therapy remains the treatment option [8]. For example, treating CRC by conducting chemotherapy causes cytotoxicity and agents resistance (e.g., 5-FU, capecitabine, cetuximab, and panitumumab) which calls for the development of more effective and novel alternative agents and/or adjuvants [9, 10]. Fortunately, melatonin is under consideration for its low toxicity and high efficacy.

Melatonin (a natural substance derived from tryptophan, and for its synthesis, refer to [11, 12]), which was initially isolated from the bovine pineal gland, shows a wide distribution from bacteria to humans [13–15]. Interestingly, melatonin also has turned out to be found in other tissues, such as lymphocytes, Harderian gland, liver, and gastrointestinal tract [16–19]. Melatonin is highly pleiotropic and regulates numerous physiological functions including circadian rhythms [20], antioxidative protection [21, 22], immune modulation [12, 23], and, with particular relevance to this article, antioncogenic and oncostatic actions [24, 25]. Given melatonin could be produced in the gastrointestinal tract, in which the total level of melatonin is ∼400 times than those in the pineal gland [26], and the protective effects of melatonin in the gastrointestinal tract (e.g., enhancing immune functions of the gut, reducing peristalsis [17], and altering intestinal microbiota community [27, 28]), and the antitumor function of melatonin, it is not surprising that melatonin could inhibit the gastrointestinal cancers including colon [29, 30]. Actually, the circadian rhythm change of blood melatonin is disordered in patients with CRC and melatonin disruption elevates the CRC incidence in humans [31, 32]. Previous studies confirmed that melatonin blocks colon carcinogenesis [33, 34]. Moreover, CGP 52608 (functions as a ligand for melatonin nuclear RZR/ROR receptor) could promote colon cancer cell apoptosis [35], and CGP 55644 (a RZR/ROR receptor antagonist) lowers the efficacy of melatonin in blocking colon tumor proliferation [36]. Altogether, these aforementioned results suggest that melatonin may inhibit CRC development and progression in humans.

Here, firstly, we summarize the cross-link between melatonin disorder and CRC occurrence; thereafter, we discuss several potential mechanisms (e.g., suppression of cancer cell proliferation, autophagy, metastasis and angiogenesis, and activation of apoptosis and cancer immunity) by which melatonin limits CRC development and progression.

## 2. Melatonin Disruption and CRC Incidence

The fluctuation of melatonin level in day and night is associated with the circadian rhythms and highly affects individual development and health [37]. Indeed, melatonin disruption is closely correlated with CRC. Epidemiologic surveys showed that the CRC incidence increased significantly in humans who have ever performed rotating shift work and/or worked at night [38–40]. Besides, Kvetnaia [41] found that the level of melatonin was increased in male patients with CRC; however, the amplitude of rhythm and secretion of melatonin in patients with CRC was significantly lowered [42, 43]. Likewise, constant illumination could cause crypt foci aberrance and promote the rodent colon cell proliferation [29]. Experimental study also reported that the melatonin concentration of serum in female rats with colon cancer was elevated compared with controls [44].

Collectively, these findings indicate that melatonin disruption is related to the elevation of CRC incidence and melatonin could be of high potential to modulate CRC development and progression.

## 3. Melatonin in CRC Cell Proliferation, Apoptosis, and Autophagy

Excessive proliferation of malignant tumors always favors in tumor progression; thus, it is meaningful to develop agents with high efficacy to inhibit CRC cell proliferation to limit CRC development and progression. The colon 38 is a transplantable adenocarcinoma originally induced in the colon of C57BL/6 mice by 1,2-dimethylhydrazine. Indeed, melatonin can inhibit murine colon 38 cancer cell proliferation [36] and reduce the multiplicity of colon tumors induced by 1,2-dimethylhydrazine (DMH) in rats [45]. Mechanistically, melatonin mainly inhibits cancer cell proliferation via (1) decreasing DNA synthesis and (2) promoting cell differentiation. It has been shown that the utilization of melatonin was significantly correlated with reduced DNA synthesis in colonic cancer cells [46, 47]. Moreover, melatonin could increase the number of highly differentiated cells to inhibit DMH-induced colon carcinoma cell proliferation [48].

The imbalance between the apoptosis and proliferation leads to malignancy development; therefore, it is another strategy to inhibit CRC development and progression by promoting cancer cell apoptosis. Actually, melatonin could induce Caco-2 cells [49] and human CRC cell apoptosis [34, 50]. 2-Hydroxymelatonin (a main melatonin metabolite in plants) could also increase CRC cell apoptosis [51]. Mechanistically, melatonin activates apoptosis through altering cell cycle program by increasing G1-phase arrest [34]. Intriguingly, it was shown that melatonin significantly contributed to 5-FU (a chemotherapeutic agent) inhibition of cell proliferation by activating apoptosis and cell cycle arrest [52]. Besides, endothelin-1 (ET-1), a peptide that serves as a survival factor in colon cancer, can promote proliferation while inhibiting apoptosis in carcinoma cells; melatonin was found to induce apoptosis by reducing ET-1 expression, thereby limiting the development and progression of colon cancer [53].

The overproliferating cancer cells compete for nutrients during the process of carcinogenesis, indicating that cancer cells may alter their metabolic states to survive. Indeed, autophagy could allow cancer cells to survive under stress (e.g., nutrients deprivation) [54]. Interestingly, melatonin can promote or inhibit autophagy (probably due to the antioxidant activity of melatonin) under specific conditions [55–58]. A series of autophagy-related proteins, such as microtubule-associated protein 1 light chain 3B (LC3B), p62, and Beclin-1, have been employed as markers of autophagy. [59] Previous study showed that melatonin treatment decreased the progression of colitis-associated colon carcinogenesis (CACC) by downregulating the process of autophagy as revealed by the expression pattern of various autophagy markers such as Beclin-1, LC3B-II/LC3B-I ratio, and p62. Melatonin intervention ameliorated inflammation and oxidative stress to inhibit autophagy, thereby blocking the progression of colitis-associated colon carcinogenesis [60].

Summarily, the inhibition of proliferation/autophagy and the activation of apoptosis could contribute to the antioncogenic effects of melatonin in inhibition of CRC.

## 4. Melatonin in CRC Metastasis, Angiogenesis, and Immunity

The cancer metastasis leads to the majority of cancer deaths because the advanced tumors are prone to invasion, migration, and metastasis, complicating the surgery and reducing its effectiveness [61, 62]. Melatonin's efficacy on migration in colonic cells has been well established. Accumulating evidence suggests that melatonin can inhibit cancer metastasis [63, 64]. It was shown that melatonin also significantly contributed to 5-FU inhibition of colon cancer cell migration [52]. Liu et al. [65] reported that melatonin decreased RKO colon cancer cell migration involving the p38/MAPK (mitogen-activated protein kinase) signaling pathway. Likewise, Zou et al. [66] also found that melatonin reduced human CRC cell proliferation and migration via the inactivation of p38 MAPK signaling. Moreover, melatonin has been suggested to decrease the depth of colon cancer invasion *in vivo* [48].

Angiogenesis serves an important role not only in physiological processes, but also in pathological conditions, including cancer [67, 68], and it favors in promoting aggressive tumor activity (e.g., tumor growth, metastasis, and invasion) [69]. Actually, the antioncogenic effects of melatonin in the suppression of CRC angiogenesis have also been investigated. Melatonin could destabilize hypoxia-inducible factor (HIF)-1*α* and/or suppress HIF-1*α* transcriptional activity in colon cancer cell [70], resulting in a reduction in the expression of vascular endothelial growth factor (VEGF), which functions as the most important angiogenesis growth factor that promotes cancer progression [71, 72]. Additionally, ET-1, a survival factor in colon cancer, is associated with the activation of angiogenesis [73]. Melatonin could also block the release of ET-1 from CRC cells, leading to inhibit angiogenesis, thereby limiting the CRC development and progression [53].

The cross-link between cancer and immune system plays a crucial role in the modulation of cancer development and progression [74, 75]. Melatonin has immune system activation property (e.g., altering macrophage and/or T-cell polarization and function) [12, 23]. Notably, circadian disturbances induce selective proinflammatory responses in the rat colonic mucosa, suggesting that melatonin may modulate cancer immunity to inhibit CRC development [76]. Indeed, melatonin is effective in restraining neoplastic growth in various tumors and cancers, including CRC, by enhancing TH cell immune response by producing interleukin (IL)-2, IL-10, and interferon-gamma (IFN-*γ*) [77]. Previous study demonstrated that melatonin exposure could decrease mitotic and apoptotic indices in the colonic adenocarcinomas and lower the expression of inflammatory mediators like nuclear factor-*κ*B (NF-*κ*B), tumor necrosis factor (TNF)-*α*, IL-1*β*, and STAT3 in the epithelial malignancies [33]. Besides, melatonin was confirmed to enhance splenic zone expansion and augment CD8^+^ lymphocytes and Fas-positive cell proliferation in DMH-induced colon carcinogenesis of rats [78].

Collectively, the published results document that melatonin blocks metastasis and angiogenesis and augments cancer immunity, thereby inhibiting CRC development and progression.

## 5. Concluding Remarks

**Figure 1:**
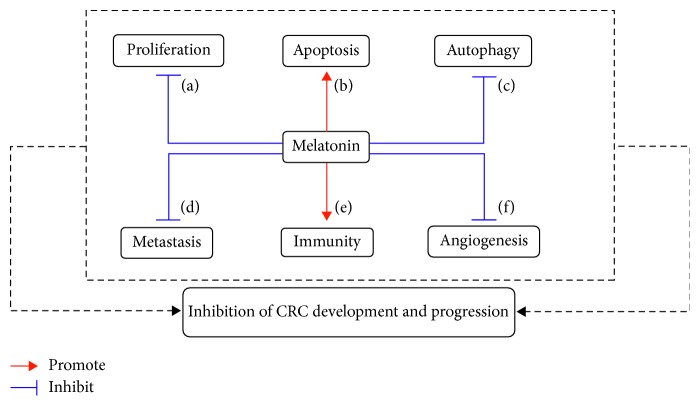
Potential mechanisms connecting to melatonin limit the development and progression of colorectal cancer (CRC). (a) Inhibiting CRC cell proliferation; (b) promoting CRC cell apoptosis; (c) reducing CRC cell autophagy; (d) blocking CRC metastasis; (e) activating CRC immunity; (f) suppressing angiogenesis.

CRC is a prevalent cancer all over the world. Melatonin disruption has been reported in patients suffering from CRC, which heralds that melatonin could be a promising agent to block CRC development and progression. Mechanistically, melatonin mainly inhibits CRC cell proliferation and autophagy, metastasis, and angiogenesis, while promoting apoptosis and enhancing cancer immunity (Figure 1). Given the mechanisms of melatonin are carried out by various other means (e.g., epigenetic modulation), and cancer development always accompany with epigenetic alteration, it is of great interest to investigate whether melatonin could inhibit CRC progression through epigenetic modification. Additionally, intestinal microbiota are closely associated with the CRC onset [79–81]; it is also interesting to study that melatonin affects CRC development that involves in shifting intestinal microbiota structure in the future.
